# Functional and Immunologic Mapping of Domains of the Reticulocyte-Binding Protein *Plasmodium vivax* PvRBP2a

**DOI:** 10.1093/infdis/jiae111

**Published:** 2024-03-05

**Authors:** Matthew Zirui Tay, Weiyi Tang, Wenn-Chyau Lee, Alice Soh Meoy Ong, Wisna Novera, Benoît Malleret, Guillaume Carissimo, Ann-Marie Chacko, Abbas El-Sahili, Julien Lescar, Yiping Fan, Rose M McGready, Cindy S Chu, Jerry Kok Yen Chan, Lisa F P Ng, Bruce Russell, François Nosten, Laurent Rénia

**Affiliations:** A*STAR Infectious Diseases Labs, Agency for Science, Technology and Research, Singapore; A*STAR Infectious Diseases Labs, Agency for Science, Technology and Research, Singapore; A*STAR Infectious Diseases Labs, Agency for Science, Technology and Research, Singapore; Department of Parasitology, Faculty of Medicine, Universiti Malaya, Kuala Lumpur, Malaysia; A*STAR Infectious Diseases Labs, Agency for Science, Technology and Research, Singapore; Laboratory for Translational and Molecular Imaging, Cancer and Stem Cell Biology Programme, Duke-NUS Medical School; Department of Microbiology and Immunology, Immunology Translational Research Programme; A*STAR Infectious Diseases Labs, Agency for Science, Technology and Research, Singapore; Infectious Diseases Translational Research Programme, Yong Loo Lin School of Medicine, National University of Singapore, National University Health System; Laboratory for Translational and Molecular Imaging, Cancer and Stem Cell Biology Programme, Duke-NUS Medical School; NTU Institute for Structural Biology, Nanyang Technological University; NTU Institute for Structural Biology, Nanyang Technological University; Department of Reproductive Medicine, KK Women's and Children's Hospital; Experimental Fetal Medicine Group, Department of Obstetrics and Gynaecology, Yong Loo Lin School of Medicine, National University of Singapore; Academic Clinical Program in Obstetrics and Gynaecology, Duke-NUS Medical School, Singapore; Shoklo Malaria Research Unit, Mahidol-Oxford Tropical Medicine Research Unit, Faculty of Tropical Medicine, Mahidol University, Mae Sot, Thailand; Centre for Tropical Medicine, Nuffield Department of Medicine, University of Oxford, United Kingdom; Shoklo Malaria Research Unit, Mahidol-Oxford Tropical Medicine Research Unit, Faculty of Tropical Medicine, Mahidol University, Mae Sot, Thailand; Centre for Tropical Medicine, Nuffield Department of Medicine, University of Oxford, United Kingdom; Department of Reproductive Medicine, KK Women's and Children's Hospital; Experimental Fetal Medicine Group, Department of Obstetrics and Gynaecology, Yong Loo Lin School of Medicine, National University of Singapore; Academic Clinical Program in Obstetrics and Gynaecology, Duke-NUS Medical School, Singapore; A*STAR Infectious Diseases Labs, Agency for Science, Technology and Research, Singapore; Department of Biochemistry, Yong Loo Lin School of Medicine, National University of Singapore; Lee Kong Chian School of Medicine, Nanyang Technological University, Singapore; Department of Microbiology and Immunology, University of Otago, Dunedin, New Zealand; Shoklo Malaria Research Unit, Mahidol-Oxford Tropical Medicine Research Unit, Faculty of Tropical Medicine, Mahidol University, Mae Sot, Thailand; Centre for Tropical Medicine, Nuffield Department of Medicine, University of Oxford, United Kingdom; A*STAR Infectious Diseases Labs, Agency for Science, Technology and Research, Singapore; Lee Kong Chian School of Medicine, Nanyang Technological University, Singapore

**Keywords:** CD98, invasion, *Plasmodium vivax*, PVRBP2a, reticulocytes

## Abstract

We previously described a novel *Plasmodium vivax* invasion mechanism into human reticulocytes via the PvRBP2a-CD98 receptor-ligand pair. Using linear epitope mapping, we assessed the PvRBP2a epitopes involved in CD98 binding and recognized by antibodies from patients who were infected. We identified 2 epitope clusters mediating PvRBP2a-CD98 interaction. Cluster B (PvRBP2a_431-448_, TAALKEKGKLLANLYNKL) was the target of antibody responses in humans infected by *P vivax*. Peptides from each cluster were able to prevent live parasite invasion of human reticulocytes. These results provide new insights for development of a malaria blood-stage vaccine against *P vivax*.

## Teaser

Here, we identify 2 clusters of epitopes mediating the functional interaction between the *Plasmodium vivax* invasion ligand PvRBP2a and its host receptor CD98. Our data reveal important epitopes for development of a *P vivax* blood-stage vaccine.

*Plasmodium vivax* causes debilitating disease in human populations, particularly in tropical and subtropical regions. After exiting from the liver stage, blood-stage merozoites of *P vivax* have a strict tropism to reticulocytes [1, 2]. Understanding the mechanisms of its entry into reticulocytes is paramount for the development of effective *P vivax* blood-stage vaccines.

While *P vivax* Duffy-binding protein has been traditionally thought to be the sole receptor mediating *P vivax* merozoite invasion [3], this parasite is now recognized to be capable of infecting individuals who are Duffy negative [4], supporting the existence of specific pathways for human reticulocyte invasion. Two additional pathways have been discovered. The first one involves the interaction of the PvRBP2b protein with transferrin receptor (CD71) [5], which is expressed on reticulocytes but not on mature red blood cells. The second pathway involves the PvRBP2a protein, which interacts with the reticulocyte-specific CD98 molecule [6].

However, the mode of interaction between PvRBP2a and CD98 remains unknown. While a negatively charged patch on PvRBP2a has been identified and hypothesized to play a role in receptor binding [7], this epitope has not been functionally confirmed. Validation of potential epitopes on PvRBP2a that may be involved in CD98 interaction is key in the development of parasite invasion–blocking treatment strategies.

In this study, we investigated the PvRBP2a epitopes involved in CD98 binding. We also determined the antigenicity of these epitopes and whether blockade of CD98 engagement of these epitopes can inhibit *P vivax* invasion. We identified 2 clusters as key players in PvRBP2a-CD98 interaction, including the previously identified patch, and confirmed their functional relevance for inhibition of parasite invasion into reticulocytes.

## METHODS

### Ethics

See supplementary methods.

### PvRBP2a_23-767_ Peptide Library

The minimal CD98-binding fragment of PvRBP2a (PVX_121920, aa23-767) was divided into an overlapping peptide library of 94 biotinylated 18-mers with 10-mer overlap. Peptides were synthesized by Mimotopes, with N-linked biotinylation and an SGSG spacer.

### CD98 Protein Production, Biolayer Interferometry, and Enzyme-Linked Immunosorbent Assay

See supplementary methods.

### Reticulocyte-Binding Assay

Fresh human cord blood was enriched for reticulocytes with CD71 microbeads and MACS columns (supplementary methods). Reticulocytes were washed twice with cold phosphate-buffered saline, added to 15 µg/mL of peptide solution, and incubated at 37°C for 1 hour. Reticulocytes were then stained with streptavidin-APC and thiazole orange at 37°C for 1 hour, washed, and acquired on a flow cytometer.

### Inhibition of *P vivax* Reticulocyte Invasion

The following were freshly prepared:

- Antibodies: anti-CD98 mouse IgG1 mAb HBJ127 [8] (Absolute Antibody) and anti-human DARC mouse IG1 mAb 2C3 [9] (Absolute Antibody); working concentration, 25 µg/mL
- Cluster A or B PvRBP2a peptides: PepLib 25, 41, 49, 52; working concentration, 100 µg/mL
- Control PvRBP2a peptides: PepLib 50, 56; working concentration, 100 µg/mL

Fresh/frozen blood isolates of *P vivax* were matured in vitro and late stages concentrated with MACS LD columns. Purified infected red blood cells were mixed with cord blood–derived CD71+ reticulocytes (sorted with CD71 beads + MACS LS columns). Infected red blood cell–reticulocyte mixtures were each mixed with an antibody/peptide as stated previously. Wells without peptide/antibody served as controls. All groups were cultivated in vitro as described before [10]. Smears were made at the start (H_0_) to ensure that ring-stage parasitemia was 0% and 24 hours later (H_24_) to determine parasitemia (10 000 red blood cells counted). Statistically significant differences were identified by 2-tailed paired *t* test with Shapiro-Wilk normality test, performed in Prism 10.1.2 (GraphPad).

## RESULTS

### Identification of CD98-Binding Epitopes in PvRBP2a_23-767_

**Figure 1.**
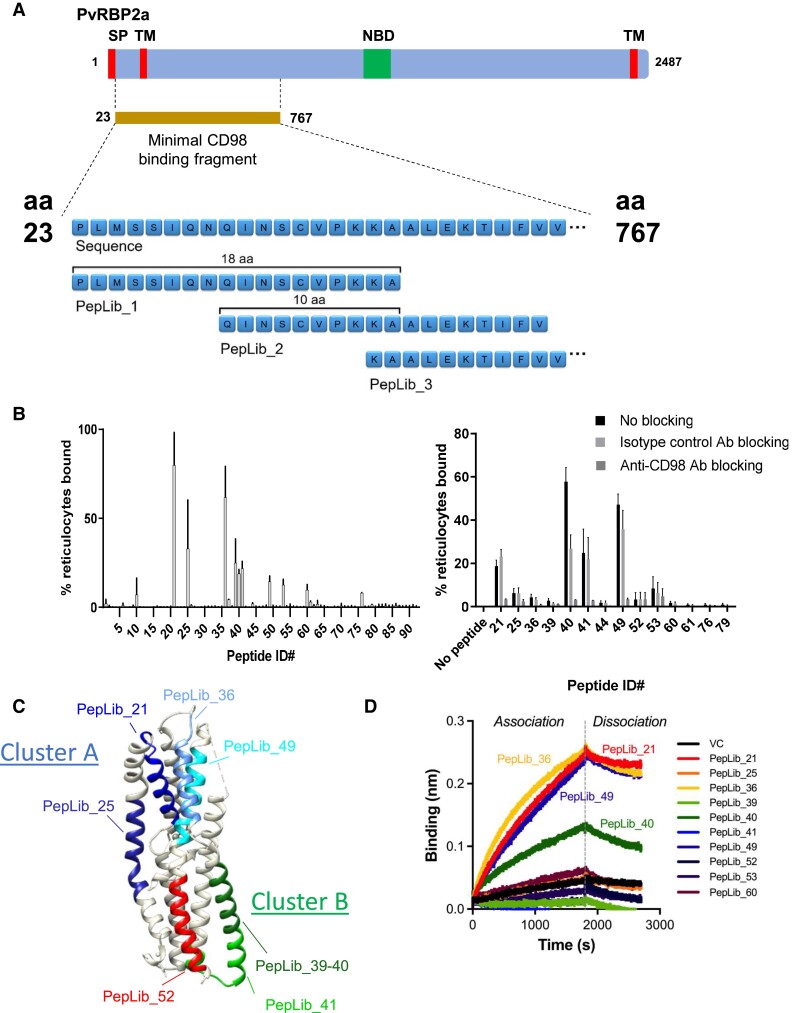
Identification and structural mapping of CD98-binding linear epitopes of PvRBP2a. *A*, The minimal CD98-binding fragment (as determined in Malleret et al [6]) is shown on a map of PvRBP2a, with putative signal peptide (SP), transmembrane domain (TM), and nucleotide-binding domain (NBD) labeled. A peptide library of 94 biotinylated 18-mers with 10-mer overlap was constructed from the minimal CD98-binding fragment, PvRBP2a_23-767_. *B*, Left: the PvRBP2a-derived peptide library was tested for binding to reticulocytes via flow cytometry. Thiazole orange–positive reticulocytes were stained with biotinylated peptides and detected with streptavidin-APC. Data are presented as mean (SD) of 2 independent replicates. Right: candidate-binding peptides were further tested to see whether their binding could be blocked by anti-CD98 antibody, showing a dependence on CD98 as the cognate ligand. Reticulocytes were blocked with the polyclonal rabbit anti-CD98 antibody (KE020) or a rabbit isotype control antibody for 15 minutes before staining with biotinylated peptide candidates and detection with streptavidin-APC. Reticulocytes were distinguished from normocytes via thiazole orange staining. Data are presented as median (range). *C*, Peptides showing immunoreactivity or CD98-binding activity are highlighted in the published 3-dimensional structure of PvRBP2a (PDB 4Z8N). PepLib_21, blue; PepLib_25, medium blue; PepLib_36, cornflower blue; PepLib_39 and PepLib_40, forest green; PepLib_41, green; PepLib_49, cyan; PepLib_52, red. *D*, Candidate-binding peptides or vehicle control (VC) was tested for direct binding to CD98 via biolayer interferometry. Biotinylated peptides (2 µM) were loaded onto streptavidin-coated sensors, which were immersed in a solution of 200nM CD98 to assess binding.

We first sought to identify the CD98-binding epitopes within the previously determined PvRBP2a_23-767_ domain, which may be responsible for CD98-binding and reticulocyte invasion function [6] (Figure 1*A*). We subdivided PvRBP2a_23-767_ into 18-mer amino acid fragments to construct an overlapping peptide library of 94 biotinylated 18-mers, with 10-mer overlap between fragments (Figure 1*A*, Supplementary Table 1). Each peptide within the PvRBP2a_23-767_ peptide library was tested for binding to fresh human reticulocytes via flow cytometry, which identified 9 peptide candidates showing binding to ≥10% of reticulocytes: PepLib_21 (PvRBP2a_183-200_), PepLib_25 (PvRBP2a_215-232_), PepLib_36 (PvRBP2a_303-320_), PepLib_39 (PvRBP2a_327-344_), PepLib_40 (PvRBP2a_335-352_), PepLib_41 (PvRBP2a_343-360_), PepLib_49 (PvRBP2a_407-424_), PepLib_53 (PvRBP2a_439-456_), and PepLib_60 (PvRBP2a_495-512_; Figure 1*B*). To confirm whether these candidates bound specifically to CD98 on the reticulocytes, the assay was repeated in the presence of anti-CD98 antibody for blocking CD98 availability (Figure 1*B*, Supplementary Figure 1A). All 9 peptide candidates exhibited reduced binding (50.7%–94.5% reduction; mean, 76.0% reduction) to reticulocytes in the presence of anti-CD98 antibody, whereas isotype control antibody did not cause reduced binding to the same degree (−24.0% to 54.2% reduction; mean, 22.5% reduction; *P* = .0007, 2-tailed paired *t* test), confirming that the binding was dependent on CD98.

While the CD98-binding peptides are not contiguous in the linear amino acid sequence of PvRBP2a, their position on the 3-dimensional protein structure of the PvRBP2a_158-455_ fragment [7] highlights that the binding peptides concentrate within 2 clusters, which we here name cluster A (PepLib_21, PepLib_25, PepLib_36, PepLib_49 peptides) and cluster B (PepLib_39, PepLib_40, PepLib_41, PepLib_53 peptides; Figure 1*C*). Cluster A corresponds to a region within the previously suggested receptor-binding site [7], especially the residues across PepLib_36. PvRBP2a has been compared with PfRh5 from *Plasmodium falciparum* given its analogous structure [7], and overlay of the PvRBP2a and PfRh5 structures [11] showed that the other linear peptides identified in cluster A are in proximity to the region corresponding to the PfRH5-binding site for basigin (the receptor for PfRh5 [12]; Supplementary Figure 2). Meanwhile, cluster B forms a novel cluster nearer the opposite end of PvRBP2a_158-455_.

To further confirm direct peptide-CD98 interactions, the binding between each candidate peptide and soluble recombinant CD98 protein was examined via biolayer interferometry (Figure 1*D*, Supplementary Figure 1B). Among the 9 candidate peptides, PepLib_21 (PvRBP2a_183-200_), PepLib_36 (PvRBP2a_303-320_), and PepLib_49 (PvRBP2a_407-424_) from cluster A and PepLib_40 (PvRBP2a_335-352_) from cluster B showed binding to soluble CD98 protein, with affinities of 54.9, 47.5, 90.3, and 70.0 nM, respectively (Supplementary Figure 1C).

### Linear Immunodominant Epitopes in PvRBP2a_23-767_

**Figure 2.**
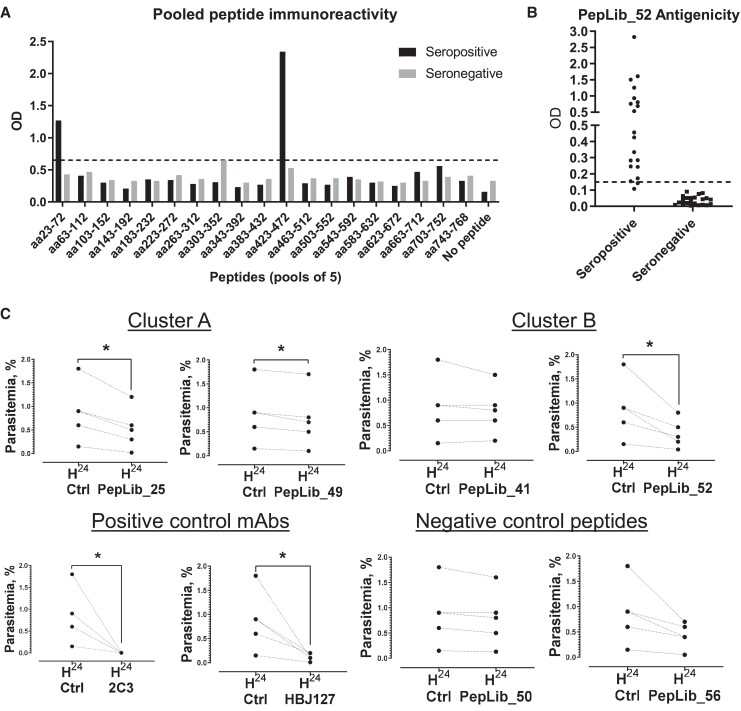
Identification of antigenic and functional linear epitopes of PvRBP2a. *A*, Peptides were pooled into groups of 5 and coated on streptavidin plates. Pooled sera were added from donors infected with *Plasmodium vivax*. Following washing, binding of peptides to donor sera was quantified with an anti-human horseradish peroxidase secondary antibody. The baseline was determined to be the mean + 3 SD of the reactivity of the seronegative sample pool to all peptide pools, and peptide pools showing a greater optical density (OD) value were deemed immunoreactive. *B*, The antigenicity of the antigenic peptide hit, PepLib_52, was confirmed by plasma from 20 donors who were seropositive and 22 who were seronegative. *C*, The effect of PvRBP2a-derived linear epitope peptides on *P vivax* invasion was examined on 5 *P vivax* clinical isolates. Selected CD98-binding PvRBP2a peptide candidates that were in cluster A (PepLib_25, PepLib_49) or cluster B/immunodominant (PepLib_41, PepLib_52), as well as negative control peptides (PepLib_50, PepLib_56) and positive control monoclonal antibodies (anti-Duffy 2C3, anti-CD98 HBJ127), were tested for their ability to inhibit *P vivax* invasion into reticulocytes. One well without addition of peptide or antibody served as a control for measurement of baseline reinvasion level. *P < .05 by 2-tailed *t* test with Shapiro-Wilk normality test.

We next sought to identify the immunodominant epitopes within the PvRBP2a_23-767_ CD98-binding domain. Groups of 5 adjacent peptides each were pooled and examined for antigenicity against a mix of sera from 20 donors infected with *P vivax* (positive by blood smear microscopy) via enzyme-linked immunosorbent assay (Figure 2*A*). Binding against at least 15 negative donors (non–*P vivax* exposed) was also examined to control for nonspecific binding. Two pools—PepLib 1-5 (representing PvRBP2a_23-72_) and PepLib 51-55 (representing PvRBP2a_423-472_)—preferentially bound to *P vivax*–positive donor sera as compared with baseline (mean + 3 SD for negative donor sera) and were thus identified to harbor antigenic epitopes. Within these pools, the individual peptides PepLib 3 (PvRBP2a_39-56_), PepLib 52 (PvRBP2a_431-448_), and PepLib 53 (PvRBP2a_439-456_) showed antigenicity against the seropositive sera mix (Supplementary Figure 3A). To confirm their antigenicity, these peptides were then examined against individual seropositive or seronegative sera. PepLib 3 and PepLib 53 were antigenic in a minority of seropositive cases (7/20 and 2/20, respectively; Supplementary Figure 3B), whereas PepLib 52 was antigenic in most seropositive cases (18/20; Figure 2*B*). Thus, we identified PepLib 52 (PvRBP2a_431-448_) as the most reactive linear B-cell epitope in the CD98-binding region of PvRBP2a. Notably, while the antigenic PepLib_52 peptide did not bind directly to reticulocytes, it lies adjacent to cluster B (Figure 1*C*), suggesting potential functional relevance for antibodies targeting this epitope.

### Sequence Diversity and Evolution in CD98-Binding and Antigenic Epitopes in PvRBP2a_23-767_

Since significant genetic variation has been found across *P vivax* isolates, we assessed whether variation was more common within clusters A and B and assessed the effects of such variation on antigenicity. We examined 612 publicly available *P vivax* PvRBP2a sequences and identified 60 protein-coding polymorphisms within the PvRBP2a_23-767_ sequence, including 11 polymorphisms with minor allele frequency >10% (Supplementary Table 2). Of these 11 polymorphisms, 5 (45.5%) were found among the cluster A and B peptides that span 150 of the 745 residues (20.1%; Supplementary Figure 3C). Notably, within the antigenic linear epitope PepLib_52 (PvRBP2a_431-448_), a G438E polymorphism was present in 28.2% of sequences. The E438 alternative peptide showed a decrease in serum binding across all donors tested, indicating that the G438E substitution decreases the antigenicity of the PvRBP2a_431-448_ epitope (Supplementary Figure 3D).

### *P vivax* Invasion Blocking Efficacy of CD98-Binding or Antigenic Linear Peptides

We next sought to verify if the linear epitopes in clusters A and B mediated functional activity, by examining their ability to block *P vivax* invasion into human reticulocytes. Two noncontiguous peptides from each cluster were tested—PepLib_25 (PvRBP2a_215-232_) and PepLib_49 (PvRBP2a_407-424_) from cluster A and PepLib_41 (PvRBP2a_343-360_) and the antigenic PepLib_52 (PvRBP2a_431-448_) from cluster B—in conjunction with 2 negative controls, PepLib_50 (PvRBP2a_415-432_) and PepLib_56 (PvRBP2a_463-480_), which do not show binding to reticulocytes (Figure 2*C*). PepLib_25 and PepLib_49 from cluster A were capable of blocking parasite invasion into reticulocytes (PepLib_25, 33.3%–86.7% [mean, 49.6%]; PepLib_49, 11.1%–33.3% [mean, 17.8%]). Surprisingly, within cluster B, the CD98-binding PepLib_41 did not inhibit parasite invasion (−33.3% to 16.7%; mean, −1.1%), but the antigenic PepLib_52 inhibited parasite invasion (44.4%–77.8%; mean, 60.2%). One of the negative control peptides surprisingly showed a trend toward inhibition (PepLib_56, 33.3%–66.7%; mean, 50%), although this did not reach statistical significance due to the large variance whereas the other negative control peptide showed no inhibition as expected (PepLib_50, 0.0%–16.7%; mean, 10.4%). Both positive monoclonal antibody controls inhibited invasion as expected (anti-Duffy 2C3, 100%; anti-CD98 HBJ127, 66.7%–94.4%; mean, 84.2%).

## DISCUSSION

In this study, we have identified 2 clusters of PvRBP2a that are involved in CD98 binding and *P vivax* invasion of reticulocytes. The identification of cluster A confirms previously reported findings that used mutagenesis and polymorphism analysis to identify regions involved in cell binding [7]. However, our study additionally identifies a second distal region (cluster B) that also contributes to binding. Notably, only cluster B contains an antigenic linear B-cell epitope, suggesting that it may be easier to target with a vaccine. Parasite invasion was decreased when peptides from cluster A or B were added as competitive decoys, supporting a functional role for both clusters. Additional studies focusing on these clusters will be important to validate CD98-binding function, especially since peptide fragments may adopt a different 3-dimensional structure relative to the full protein.

Comparison of the PvRBP2a structure with the structurally conserved PfRh5 revealed that cluster A is in proximity to the ligand-binding region in the case of PfRh5's binding model to basigin (Supplementary Figure 2). It remains unclear how cluster B is involved in ligand binding. However, other members of the RBP family with domains that are structurally similar to PvRBP2a_23-767_ can bind ligands using very different binding sites (eg, PvRBP2b with side-on binding to CD71); thus, it is clear that this domain can be utilized in multiple ways for host receptor engagement. In the case of CD98 binding to PvRBP2a, it is possible that binding at both sites occurs simultaneously or that a multiple-step mode of binding occurs with different epitopes engaged at each step.

Polymorphisms were overrepresented in clusters A and B, suggesting active selection and evolution at these regions. The E304K mutation at cluster A has been reported to increase erythrocyte binding, whereas the G438E polymorphism decreased erythrocyte binding [7]. Here, we show that the G438E polymorphism in cluster B decreased antigenicity, which suggests that the E allele may facilitate immune escape at a cost to host cell engagement. There remains a critical need for an efficacious vaccine against *P vivax*. Here, we identify novel functional epitopes on an important *P vivax* antigen, PvRBP2a, to help elucidate its interaction mechanism with its host ligand CD98. At the same time, directly blocking host ligand interaction may not be the only way to neutralize an invasion pathway. For instance, in the case of the *P falciparum* invasion ligand AMA-1, many potent monoclonal and polyclonal antibodies do not block AMA-1 interaction with its host ligand RON2L but instead show other mechanisms, including blocking of secondary processing and ligand redistribution [13, 14]. Further studies to elucidate the most effective mechanisms for targeting the important PvRBP2a invasion pathway continue to be needed. With the current study, these will provide new insights for development of a blood-stage vaccine against *P vivax*.

## Supplementary Data

Supplementary materials are available at *The Journal of Infectious Diseases* online (http://jid.oxfordjournals.org/). Supplementary materials consist of data provided by the author that are published to benefit the reader. The posted materials are not copyedited. The contents of all supplementary data are the sole responsibility of the authors. Questions or messages regarding errors should be addressed to the author.

## Supplementary Material

jiae111_Supplementary_Data

## Notes

***Acknowledgments.*** We are indebted to the staff of the Mae Sot Malaria Clinic and the clinics associated with the Shoklo Malaria Research Unit, as well as the patients attending these clinics. This publication uses data from the MalariaGEN *P falciparum* Community Project (www.malariagen.net/projects/p-falciparum-community-project), as described by Pearson et al [15]. Genome sequencing was performed by the Wellcome Trust Sanger Institute, and the community project is coordinated by the MalariaGEN Resource Centre with funding from the Wellcome Trust (098051, 090770). We thank the Agency for Science, Technology and Research (A*STAR)’s Singapore Immunology Network (SIgN) Flow Cytometry Platform for enabling the flow cytometry work for this publication.

***Author contributions*.** M. Z. T., W. T., and L. R. conceived and designed the study. M. Z. T., W. T., A. S. M. O., G. C., and A. E.-S. performed antigenicity mapping and reticulocyte-binding characterization. W.-C. L. and A. S. M. O. performed invasion inhibition characterization. W. N. and A.-M. C. designed and performed biolayer interferometry analyses. Y. F., R. M. M., C. S. C., J. K. Y. C., B. R., and F. N. collected and processed patient and parasite samples. B. M., A.-M. C., J. L., J. K. Y. C., B. R., F. N., L. F. P. N., and L. R. provided scientific direction and supervision. M. Z. T. and L. R. wrote the original draft. All authors critically reviewed and edited the manuscript. All authors approved the final version of the manuscript.

***Disclaimer.*** The funders had no role in study design, data collection and analysis, the decision to publish, or preparation of the manuscript.

***Financial support.*** This work was supported by core funding from the Agency for Science, Technology and Research to A*STAR Infectious Diseases Labs, by a Start-up University Grant from the Ministry of Education–Singapore (022388-00001), and by Temasek Foundation (grant “Temasek Foundation Infectious Diseases Programme for Surveillance and Diseases X Resilience”) to L. R.; by the Singapore National Medical Research Council IRG Grant (NMRC/OFIRG/0065/2018) to L. R. and J. L.; by a National Medical Research Council STaR Investigator Award (NMRC STaR22jul-0004) to J. K. Y. C.; by a Marsden Grant Award (RGUOO180301 to L. R. and B. R.); by The Wellcome Trust of Great Britain, as part of the Oxford Tropical Medicine Research Programme of Wellcome Trust–Mahidol University, to the Shoklo Malaria Research Unit; by Singapore's Health and Biomedical Sciences Industry Alignment Fund Pre-positioning (grant H18/01/a0/018 to W. N. and A.-M. C.) administered by the Agency for Science, Technology and Research; and by research grants from the Biomedical Research Council and the National Research Foundation Singapore Immunomonitoring Service Platform (ISP: NRF2017_SISFP09) to A*STAR’s Sign Flow Cytometry Platform.
