# Supplementary material for: Functional and Immunologic Mapping of Domains of the Reticulocyte-Binding Protein Plasmodium vivax PvRBP2a
Source: J Infect Dis. 2024 Mar 5;230(3):e737–42. doi: 10.1093/infdis/jiae111 (PMC11420707; doi:10.1093/infdis/jiae111)
Supplement: jiae111_Supplementary_Data [file jiae111_supplementary_data.zip › PvRBP2a_SupplementaryFigureLegends.docx]

**Supplementary Figure 1. PvRBP2a epitopes for reticulocyte and CD98 binding.** (A) Candidate binding peptides were further tested to see whether their binding could be blocked by anti-CD98 antibody, showing a dependence on CD98 as the cognate ligand. Reticulocytes were blocked with the polyclonal rabbit anti-CD98 antibody (KE020) or a rabbit isotype control antibody for 15 minutes before staining with biotinylated peptide candidates and detection with streptavidin-APC. Reticulocytes were distinguished from normocytes via thiazole orange staining. Representative flow cytometry plots are shown for PepLib_49 binding to reticulocytes (top left), PepLib_49 binding in the presence of rabbit isotype control antibody (top middle), or PepLib_49 binding in the presence of rabbit anti-CD98 antibody KE020 (top right), as well as negative control PepLib_76 binding to reticulocytes (bottom left), Thiazole Orange-only staining (bottom middle), and unstained (bottom right) conditions. (B) Candidate binding peptides were tested for direct binding to CD98 via biolayer interferometry. 2µM biotinylated peptides were loaded onto streptavidin-coated sensors, which was immersed in a solution of 200nM CD98 to assess binding. (C) Peptides with detectable binding in the initial binding screen were further analyzed for their binding kinetics to CD98 via biolayer interferometry, with 3 independent replicates. The mean and standard error for dissociation constant (K_D_), on-rate (k_a_) and off-rate (k_d_) are given.

**Supplementary Figure 2. Overlay of PfRH5 on the PvRBP2a structure.** Structure of PfRh5 (light grey, 50% transparent) and bound basigin (light pink) (PDB 4U0Q) overlaid on PvRBP2a structure (white, PBD 4Z8N). Peptides showing immunoreactivity or CD98-binding activity are highlighted in the PvRBP2a structure. Colors: PepLib_21: Blue; PepLib_25: Medium blue; PepLib_36: Cornflower blue; PepLib_39 and PepLib_40: Forest Green; PepLib_41: Green; PepLib_49: Cyan; PepLib_52: Red.

**Supplementary Figure 3. Identification of immunodominant linear epitopes of PvRBP2a.** (A) Of the two peptide pools showing immunoreactivity, individual peptides were tested to identify immunoreactive linear epitopes in PvRBP2a. (B) The antigenicity of the three peptide hits, PepLib_3, PepLib_52 was determined using plasma from 20 individual seropositive donors. (C) A schematic of the PvRBP2a23-767 sequence is shown, with polymorphisms above 10% minor allele frequency in the PvRBP2a23-767 region marked in white, and corresponding pie charts showing the allele frequency (orange: wild type allele frequency, blue: alternate allele frequency). Segments corresponding to peptides showing immunoreactivity or CD98-binding activity have been colored – PepLib_21: Blue; PepLib_25: Medium blue; PepLib_36: Cornflower blue; PepLib_39 and PepLib_40: Forest Green; PepLib_41: Green; PepLib_49: Cyan; PepLib_52: Red; PepLib_60: Brown. Polymorphisms which lie within these regions are bolded. (D) The effect of a known SNP (G438E) in immunoreactive peptide PepLib_52 on its immunoreactivity was examined among the seropositive donors.
