# Supplementary material for: Functional and Immunologic Mapping of Domains of the Reticulocyte-Binding Protein Plasmodium vivax PvRBP2a
Source: J Infect Dis. 2024 Mar 5;230(3):e737–42. doi: 10.1093/infdis/jiae111 (PMC11420707; doi:10.1093/infdis/jiae111)
Supplement: jiae111_Supplementary_Data [file jiae111_supplementary_data.zip › PvRBP2a_SupplementaryMethods_20231214_clean.docx]

**Supplementary Data**

**Functional and immunological mapping of domains of the reticulocyte binding protein, Plasmodium vivax PvRBP2a**

**Authors:** Matthew Zirui Tay, Weiyi Tang, Wenn-Chyau Lee, Alice Soh Meoy Ong, Wisna Novera, Benoît Malleret, Guillaume Carissimo, Ann-Marie Chacko, Abbas El-Sahili, Julien Lescar, Yiping Fan, Rose M McGready, Cindy S Chu, Jerry Kok Yen Chan, Lisa FP Ng, Bruce Russell, François Nosten, Laurent Rénia

**Online methods**

**Ethics**

Written informed consent was obtained from all participants and participants’ parents or legal guardians (parental consent for age under 17) and study was conducted according to Declaration of Helsinki principles. Human cord blood and healthy adult plasma samples were collected under SingHealth CIRB 2019/2443- and 2017/2806-approved protocols respectively. Plasma samples from febrile *P. vivax*-infected patients (positive by microscopy) from Mae Sot, Thailand, were collected and tested in accordance with protocols approved by the University of Oxford Tropical Research Ethics Committee (OXTREC 17-11) and the Ethics Committee of the Faculty of Tropical Medicine at Mahidol University (MUTM 2008-215). Written informed consent was received before participation. Patient demographics are given in Supplemental Table 3. *P. vivax*-infected samples were collected in Shoklo Malaria Research Unit (SMRU) under approved ethics: OXTREC 04–10 (University of Oxford, UK); TMEC 09–082 (Ethics Committee, Faculty of Tropical Medicine, Mahidol University, Thailand).

Animal experiments were approved by the A*STAR Institutional Animal Care and Use Committee in accordance with the rules and regulations of the Singaporean Agri-Food and Veterinary Authority and the National Advisory Committee for Laboratory Animal Research.

**CD98 cloning and expression in Sf-9 cells**
The coding sequence of the soluble domain of CD98 (W218-A630) was cloned using the primers A 5′-TATCCACCTTTACTGTTAGGCCGCGTAGG-3′ and B 5′-TACTTCCAATCCATGTGGTGGCACACGGGC-3′, and the amplified fragment was purified before insertion into a pFastBac-LIC-BseR1 (Addgene) plasmid using a Gibson Assembly Cloning kit (NEB). Escherichia coli Top10 competent cells were transformed with the ligated plasmid (pFastBac-CD98) and spread on LB plates supplemented with ampicillin. E. coli DH10Bac competent cells were transformed with the purified pFastbac-CD98 plasmid. Extraction of the bacmid and virus packaging was done according to Bac-to-Bac (Invitrogen). For expression, Sf-9 cells were infected at a multiplicity of infection of 1 for 72 h at 27 °C under agitation. Cells were collected at 3,000 × g for 15 min, and the cell pellet was stored at -20 °C.

**CD98 purification**
Cells were resuspended in 20 mM Tris pH 7.5, 500 mM NaCl and 10 mM imidazole and lysed by sonication. After centrifugation at 20,000 × g for 45 min, the filtered supernatant was injected into a nickel affinity column (HisTrap 1 ml, GE Healthcare). After a washing step of 6% of 20 mM Tris-HCl pH 7.5, 500 mM NaCl and 500 mM imidazole (buffer B), the protein was eluted with 100% of buffer B and injected into a Superdex 75 Hiload 26/60 gel-filtration column (GE Healthcare) equilibrated in PBS. The protein fractions were pooled and concentrated before being flash-frozen in liquid nitrogen and stored at -80 °C.​

**CD98 binding biolayer interferometry**

Binding sensorgrams were collected on the Octet Red 96e system. Fresh streptavidin sensors were used without any regeneration step. Streptavidin sensor, without biotinylated peptide loading, but with maximum concentration of non-biotinylated CD98 was used as the reference. Kinetic binding assays were performed using Octet Data Acquisition version 10.0.1.3 at 37°C with orbital shaking speed of 1,000 rpm. The assay was carried out in PBS buffer supplemented with 0.05% Tween-20 and 1mg/mL BSA (PBST/BSA). For initial screening, the biosensor was sequentially dipped into wells containing 200μL of solution each: i) baseline, PBST/BSA (30 s), ii) loading, 2µM biotinylated peptide fragment or vehicle control (VC) (5 min), iii) washing, PBST/BSA (30 s), iv) blocking, 25μM biocytin (5 min) to minimize non-specific binding of CD98, v) washing, PBST/BSA (30 s), vi) association, 200 nM non-biotinylated CD98 (30 min) and vii) dissociation, PBST/BSA (15 min). Detailed kinetic binding parameters of CD98 with lead binding peptides were similarly assessed, except using varying concentrations of non-biotinylated CD98 with 30 min association, followed by 30 min dissociation. Binding sensorgrams were analysed using ForteBio’s Data Analysis version 10.0, where reference sensor was subtracted from all sensorgrams and aligned to the baseline. Normalized sensorgrams were globally fit to a 1:1 Langmuir binding model. Association (*k_a_*, M^−1^s^−1^), dissociation (*k_d_*, s^−1^), and (K_D_, nM) affinity constants were calculated based on quadruplicate runs and are represented as mean ± S.E.M.

**Reticulocyte enrichment**

Reticulocytes were enriched from the human cord blood through the selection of CD71+ reticulocytes, using human CD71 microbeads (Miltenyi Biotec, clone AC108.1). Around 1-2ml of blood at 50% haematocrit in PBS was passed through a LS column. After two washing steps using PBS, the reticulocytes were stained with reticulocyte stain (Sigma Aldrich New Methylene Blue) and viewed under the microscope to ensure >80% purity of reticulocytes after selection.

**Sequence Alignments**

612 publicly available *P. vivax* PvRBP2a sequences [PlasmoDB[1] and MalariaGEN (www. https://www.malariagen.net)] were utilized for SNP identification. Minor allele frequency was considered to be >10% only if both the reference (SalI) and alternate allele exceeded 10% frequency in the dataset.

**Protein structure comparison**

The RCSB Protein Data Bank (PDB) structures for PvRBP2b and PfRh5 (4U0Q[2] and 6D03[3] respectively) were overlaid on the PvRBP2a structure (4Z8N[4]) using the MatchMaker tool in Chimera[5]. Structural figures were prepared using Chimera.

**ELISA**

Streptavidin-coated plates (Pierce) were blocked with 1% casein/1% BSA in 0.1% PBST overnight at 4°C, then coated with blocked with PvRBP2a-derived biotinylated peptides at 1:1000 dilution (approximately 15ug/ml) in 0.2% casein/0.2% BSA at room temperature for 1 hour. Pooled or individual donor sera was then applied at 1:267 (pooled) or 1:1000 (individual) dilution in 0.2% casein/0.2% BSA at room temperature for 1 hour, followed by secondary goat anti-mouse IgG-HRP at 1:1000 dilution in 0.1% casein/0.1% BSA at room temperature for 1 hour, then TMB substrate (Surmodics, BioFX® TMB One Component HRP Microwell Substrate) incubation for 5 minutes.

References

1. PlasmoDB: An integrative database of the Plasmodium falciparum genome. Tools for accessing and analyzing finished and unfinished sequence data. The Plasmodium Genome Database Collaborative. Nucleic Acids Res **2001**; 29:66-9.

2. Wright KE, Hjerrild KA, Bartlett J, et al. Structure of malaria invasion protein RH5 with erythrocyte basigin and blocking antibodies. Nature **2014**; 515:427-30.

3. Gruszczyk J, Huang RK, Chan LJ, et al. Cryo-EM structure of an essential Plasmodium vivax invasion complex. Nature **2018**; 559:135-9.

4. Gruszczyk J, Lim NT, Arnott A, et al. Structurally conserved erythrocyte-binding domain in Plasmodium provides a versatile scaffold for alternate receptor engagement. Proc Natl Acad Sci U S A **2016**; 113:E191-200.

5. Pettersen EF, Goddard TD, Huang CC, et al. UCSF Chimera--a visualization system for exploratory research and analysis. J Comput Chem **2004**; 25:1605-12.
