# Supplementary figures and images for: Functional and Immunologic Mapping of Domains of the Reticulocyte-Binding Protein Plasmodium vivax PvRBP2a
Source: J Infect Dis. 2024 Mar 5;230(3):e737–42. doi: 10.1093/infdis/jiae111 (PMC11420707; doi:10.1093/infdis/jiae111)

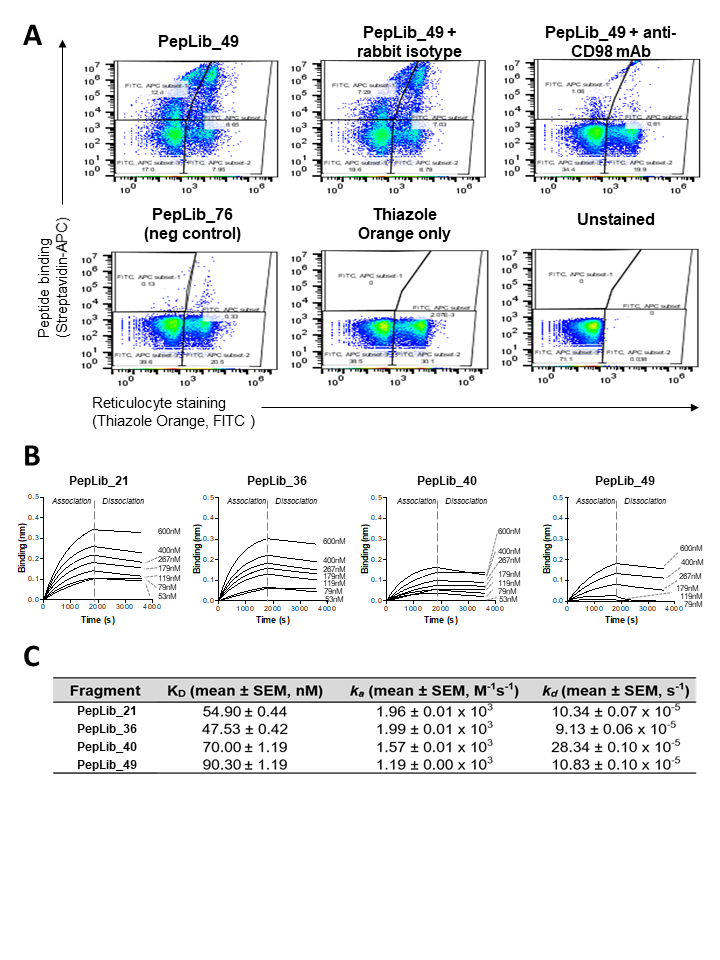

Supplement: jiae111_Supplementary_Data [file jiae111_supplementary_data.zip › SupplementaryFigure1_20231205.TIF]

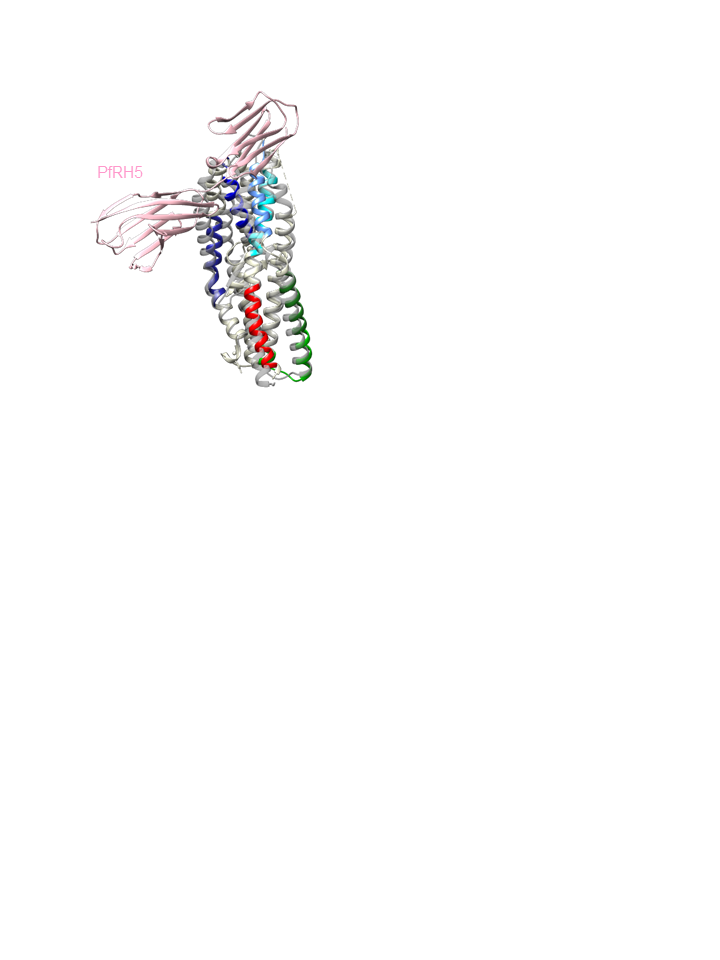

Supplement: jiae111_Supplementary_Data [file jiae111_supplementary_data.zip › SupplementaryFigure2_20231205.TIF]

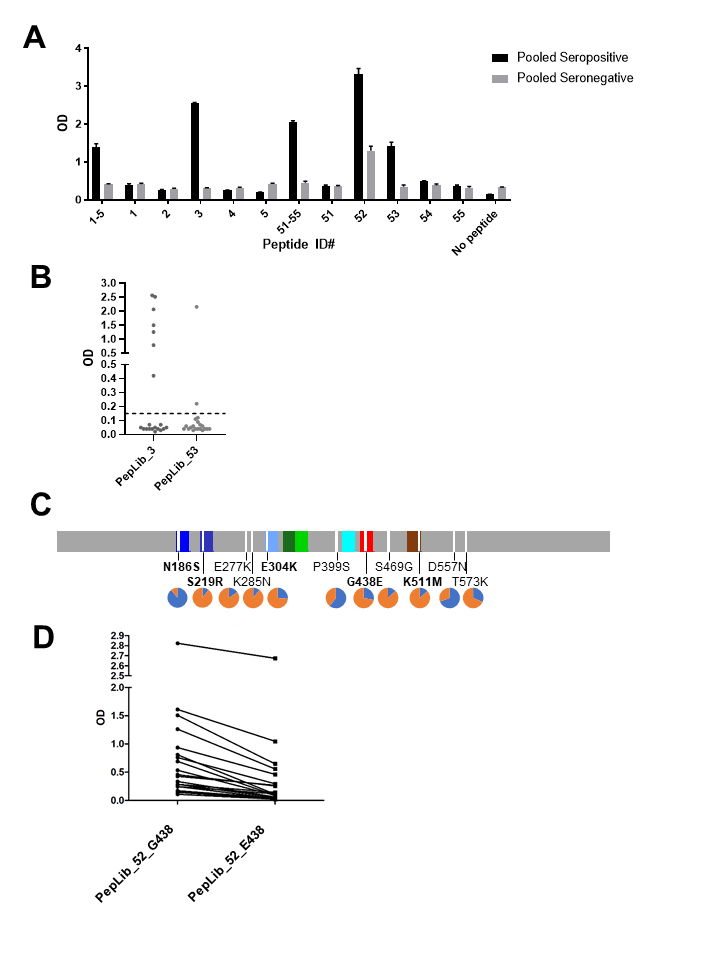

Supplement: jiae111_Supplementary_Data [file jiae111_supplementary_data.zip › SupplementaryFigure3_20231205.TIF]
